# An e-Health Psychoeducation Program for Managing the Mental Health of People with Bipolar Disorder during the COVID-19 Pandemic: A Randomized Controlled Study

**DOI:** 10.3390/jcm13123468

**Published:** 2024-06-14

**Authors:** Alessandra Perra, Federica Sancassiani, Elisa Cantone, Elisa Pintus, Silvia D’Oca, Alessio Casula, Sara Littarru, Sara Zucca, Davide Tumolillo, Irene Pinna, Diego Primavera, Giulia Cossu, Antonio Egidio Nardi, Goce Kalcev, Mauro Giovanni Carta

**Affiliations:** 1 Department of Medical Sciences and Public Health, University of Cagliari, Cittadella Universitaria, S.P. Monserrato-Sestu km 0,700, 09042 Cagliari, Italy; alessandra.perra@unica.it (A.P.); federicasancassiani@yahoo.it (F.S.); elisa.cantone@libero.it (E.C.); pintuselisa@yahoo.it (E.P.); silvia.doca13@gmail.com (S.D.); alessio.casula8@gmail.com (A.C.); littarrusara@gmail.com (S.L.); sarazucca3@gmail.com (S.Z.); davedaddo@gmail.com (D.T.); irene.pinna88@outlook.it (I.P.); diego.primavera@unica.it (D.P.); giuliaci@hotmail.com (G.C.); maurogcarta@gmail.com (M.G.C.); 2 Institute of Psychiatry, Federal University of Rio de Janeiro, Rua Visconde de Piraja 407/702, Rio de Janeiro 21941-972, Brazil; antonioenardi@gmail.com

**Keywords:** psychiatric rehabilitation, bipolar disorders, recovery, COVID-19, quality of life, biorhythms

## Abstract

**Background:** Social rhythm dysregulation has been identified as a determining factor in bipolar disorder (BD) relapses. It directly impacts individuals’ quality of life (QoL). This study aims to present preliminary data on the efficacy of an e-health psychoeducational intervention for BD for improving clinical outcomes. **Methods:** This study used an open-label, crossover, randomized controlled trial design. The inclusion criteria consisted of a BD diagnosis, affiliation with the Consultation Psychiatry and Psychosomatic Center at the University Hospital in Cagliari, Italy, age over 18, and the obtaining of informed consent. Anxiety and depressive symptoms, QoL, and social and biological rhythms were measured using standardized instruments validated in Italian. **Results:** A total of 36 individuals were included in the experimental group (EG) and 18 in the control group (CG). The final sample consisted of 25 in the EG and 14 in the CG. A statistically significant improvement in QoL was found in the EG post-treatment (*p* = 0.011). Significant correlations were found between QoL and the dysregulation of biorhythms in the EG at T0 (*p* = 0.0048) and T1 (*p* = 0.0014). **Conclusions:** This study shows that, during extreme distress, an e-health group psychoeducation intervention for people with BD could significantly improve the perception of QoL. The results must be confirmed by studies conducted with larger-sized samples.

## 1. Introduction 

The COVID-19 pandemic had an intense impact on individuals and communities worldwide [1,2]. People experienced distress due to the direct consequences of the SARS-CoV-2 disease [3]. These direct consequences included the illness and death of friends and relatives, fear of contagion, discomfort associated with quarantine, and the resulting economic and social instability [3,4]. Social isolation, anxiety, fear of contagion, uncertainty, chronic stress, and economic difficulties have contributed to the onset or exacerbation of depression, anxiety, substance use, and other psychiatric disorders, especially among vulnerable populations living in areas with high COVID-19 prevalence [5,6,7]. This risk has also been found to be higher among healthcare workers [8,9,10] and individuals with chronic diseases and disabilities, particularly those already affected by mental illnesses [11]. Various risk factors have increased the vulnerability of these populations. First, the pandemic has intensified the consequences of loneliness and social isolation, leading to the loss of contact with mental health professionals and caregivers [12]. Additionally, the increased severity of potential infections has been linked to concurrent health conditions, such as obesity and metabolic diseases, and a strong correlation with cardiac and respiratory disorders [13]. People with psychosocial disabilities have demonstrated elevated rates of infection and increased risk of mortality if infected [14].

People with bipolar disorder (BD) have shown specific vulnerability during the COVID-19 pandemic, particularly due to strict lockdowns [15]. These measures may have disrupted personal, social, and biological rhythms, which are crucial in managing BD [16].

In general, individuals with BD experience alternating phases of depression, hypo/manic episodes, and periods of euthymia [17]. These fluctuations, along with cognitive deficits, can lead to substantial impairments in overall functioning and a diminished quality of life [18]. Based on the World Health Organization’s World Mental Health surveys, BD is regarded as the second most debilitating illness in terms of days lost due to functional impairment [19].

The dysregulation of biological rhythms is a known trigger for crises in bipolar spectrum disorders [20]. Moreover, biological rhythms significantly impact individuals’ quality of life (QoL) [21,22,23]. Additionally, it has been suggested that the subclinical dysregulation of social and biological rhythms could serve as a common risk factor for various psychiatric disorders, including BD [24]. According to this perspective, BD may thus result from the convergence of hyperactivity and rhythm dysregulation [25,26]. During the pandemic, the dysregulation of social rhythms, linked to lockdown measures, was associated with the onset of mood disorder episodes, also affecting QoL [15,24]. Amplified fear during the pandemic, driven by a lack of knowledge and a sense of losing control, further compounded these issues [27]. 

This context emphasizes the need to support the psychological well-being of particularly vulnerable populations, such as people with psycho-social disabilities, during a pandemic [28]. Specifically, for individuals with BD, a crucial intervention should involve providing information about pandemic-related risks and the additional challenges linked to living with BD, including depressive relapses, the dysregulation of biological and social rhythms, and related conditions, such as obesity and co-morbid disorders [29]. 

Psychoeducational interventions in mental health encompass structured programs that integrate psychoeducational information with cognitive–behavioral therapy techniques. These interventions aim to empower individuals with mental health challenges to better understand their disorder, manage their symptoms, and enhance their overall QoL [30,31]. Such programs typically cover various topics, including the nature of the disorder, treatment adherence, stress management strategies, and problem-solving techniques [32]. The goal is to provide individuals and their families with the requisite knowledge and skills necessary to face the daily challenges associated with mental health disorders [33]. A psychoeducational intervention is one of several psychosocial interventions that adheres to the evolutionary model of social determinants [34], in which strategies are improved upon to enable individuals to achieve their personal goals in everyday life [35]. During the COVID-19 pandemic, the significance of psychoeducational interventions for managing stress and anxiety related to the health crisis was underscored [36]. These programs have been adapted for delivery through digital platforms (e-health), allowing for remote access despite social restrictions [37]. Psychoeducational programs targeting pandemic management have emphasized strategies for coping with health-related anxiety, addressing the challenges of social isolation, and promoting preventive behaviors, such as mask-wearing and social distancing [38]. Consistent with this evidence, psychoeducation should also address issues related to the dysregulation of rhythms and the management of hyperactivity/exploratory behavior during periods of restriction, such as lockdowns [39]. Indeed, psychoeducation is an effective psychosocial intervention used to manage BD [40]. To achieve this objective in this complex context, this study employed remote intervention tools (telemedicine/telerehabilitation), which have been previously used in many mental healthcare settings [41,42,43]. 

The aim of this study is to report preliminary data on the improvement of clinical outcomes (QoL, biological rhythms, anxiety, and depressive symptoms) and the correlations between QoL and biorhythms after an e-health psychoeducational intervention supporting people with BD. 

## 2. Methods 

### 2.1. Design

This study employed an open-label, crossover, randomized controlled trial design, adhering to the reporting guidelines outlined in the CONSORT extension for randomized clinical trials [44].

The crossover study design was required for this study’s implementation within a healthcare setting during the COVID-19 pandemic. Given the importance of a psychoeducational intervention for patients with BD in light of the pandemic’s consequences, the limitation of having only one healthcare team, and the necessity to intervene in two groups at different times made a crossover design suitable to address these needs. Group A, consisting of those immediately receiving the treatment in addition to their usual care, was compared to Group B, who served as a control and received only their usual care during the same period. Subsequently, Group B underwent the experimental intervention, and participants from both Group A (in the first phase) and Group B (in the second phase) were combined in this latter experimental group.

### 2.2. Sample, Recruitment, and Allocation

This study’s sample consisted of patients of the Consultation Psychiatry and Psychosomatic Center at “San Giovanni di Dio” University Hospital (Cagliari, Italy) during the third and fourth waves of the pandemic (May–November 2021), when restrictive measures were in place. This study was developed to address the needs identified among patients following an assessment of the lockdown’s impact during the first and second waves of the pandemic (February 2020/May 2020–October/December 2020), as previously published elsewhere [15]. Those who met the inclusion criteria were contacted again to sign the informed consent and resolve any issues related to the technologies needed to attend the psychoeducation (i.e., internet access, having a personal computer or a smartphone, downloading the Zoom app). The inclusion criteria consisted of a diagnosis of BD according to the *Diagnostic and Statistical Manual of Mental Disorders, Fifth Edition* (DSM-V) [45], affiliation with the Consultation Psychiatry and Psychosomatic Center at “San Giovanni di Dio” University Hospital (Cagliari, Italy), being over 18 years old, and providing informed consent. The exclusion criteria included refusal to participate in this study, serious medical conditions that precluded participation in telerehabilitation interventions, and failure to meet the general inclusion criteria. The eligible individuals were randomly assigned to one of two groups with an allocation ratio of 1:1. The random allocation sequence was generated through a computer-generated randomization list administered by the University of Cagliari, Italy. The randomization process was conducted by a biometrician who remained blinded to the participants’ identities and was not involved in either the assessment or the subsequent analysis. Blinding was not feasible for both the participants and the mental health workers involved in the project, due to the characteristics of the intervention.

### 2.3. Outcomes and Instruments 

The sociodemographic information of the participants was collected through an ad hoc data sheet. All the other instruments were validated in the Italian language. The questionnaires were administered at the beginning, pretreatment (T0), corresponding to the third phase of restrictive measures in Italy (March–May 2021), and at the end of the psychoeducation program (T1). Due to the crossover design, the first group’s evaluation ended in July 2021 and the second group’s evaluation ended in November 2021, representing the fourth phase. 

Anxiety symptoms were assessed using the seven-item Generalized Anxiety Disorder (GAD-7), a self-administered questionnaire that evaluates these symptoms, independently of the diagnosis of an anxiety disorder. In this study, a GAD score > 7 was considered an indicator of clinically significant anxiety symptoms [46]. The GAD-7 has demonstrated strong reliability and construct validity, as indicated by a Cronbach’s alpha of 0.88 [47].

Depressive symptoms were assessed using the Hamilton Depression Rating Scale (HAM-D). It consists of 21 items, and the total score allows for the assignment of severity scores for the symptoms. In this study, a HAM-D total score > 20 was considered an indicator of clinically significant depressive symptoms [48]. It has demonstrated good reliability and validity, with a Cronbach’s alpha of 0.80 [49,50].

QoL was assessed using the Short Form Health Survey (SF-12), a self-administered questionnaire consisting of 12 items that assess two dimensions: physical health and mental health [51]. In this study, the mean ± 1 SD of the Italian normative sample [52] was considered as the cut-off score to establish poor/good QoL. It has been shown to be a valid and reliable instrument, with a Cronbach’s alpha of 0.69 [53]. 

The regularity of biological rhythms was measured using the Biological Rhythms Interview of Assessment in Neuropsychiatry (BRIAN), which is an interview consisting of 18 items that investigate four main areas related to the dysregulation of biological rhythms: sleeping, activity, social rhythms, and eating [54]. In this study, the mean ± 1 SD of the Italian normative sample [54] was considered as the cut-off score to establish the poor/good regulation of rhythms. It has good validity and reliability, with a Cronbach’s alpha of 0.80 [55].

### 2.4. Experimental and Control Group Intervention 

A specific psychoeducational model was developed for the experimental group involving individuals with BD during the initial waves of the pandemic. This intervention, conducted in a group setting, was delivered online using telemedicine/telerehabilitation technology tools (internet connection, Zoom app). The e-health psychoeducation intervention adopted the same logical framework in comparison to existing, effective traditional psychoeducational models [40,56,57]. The overall objective was to provide information about the clinical condition and symptoms, while identifying effective coping strategies to manage mental health and reduce the clinical impact of the pathology. Specifically, for BD, psychoeducational treatments focus on relapse prevention by providing individuals with strategies to recognize early warning signs related to symptoms and lifestyle dysregulation [58]. 

The primary goal of this specific e-health psychoeducation intervention was to reduce the negative effects associated with the COVID-19 pandemic and the implemented restrictive measures (lockdown), such as depressive and anxiety symptoms and biological dysregulation, all of which are crucial in the management of BD. The intervention consisted of one session per week for two months, totaling eight sessions, each lasting 90 min. The sessions were facilitated by psychologists, psychiatric rehabilitation technicians, and professional health educators. The sessions were structured by topic and included the following: a session on the introduction to psychoeducation, with objectives and participation modalities; a session on the effects of the pandemic on physical and mental health; one session on stress and coping strategies; a session on anxiety symptoms and coping strategies; a session on depressive symptoms and coping strategies; a session on biological rhythm dysregulation and coping strategies; and two sessions for final feedback, with a focus on acquired strategies for daily life. All sessions included an informative section (15 min); the identification of personal strategies (20 min); learning through commonly used techniques, such as shaping, scaffolding, or role-playing (45 min); and finally, a section with home assignments identified as recovery goals (10 min). In this context, a salutogenic, personal, recovery-oriented model was promoted [56,59,60], emphasizing strategies to manage the effects of the pandemic in daily life, and aligning with participants’ personal goals and the specific needs of the target population. 

The control group can be considered inactive as they remained on a waiting list, receiving only usual care consisting of psychiatric visits and pharmacological treatment.

### 2.5. Statistical Analysis

The data were analyzed using IBM SPSS Statistics 25. In all the statistical tests, a *p*-value < 0.05 was considered statistically significant. Frequencies (percentages) or the mean ± standard deviation were used for the descriptive statistics about sociodemographic and clinical variables. The homogeneity between the experimental and control groups regarding sociodemographic variables was tested by the χ^2^ test or one-way ANOVA for the categorical or dimensional variables.

The comparison of frequencies of the scores of the HAM-D, SF-12, BRIAN, and GAD-7 questionnaires at the beginning, or pretreatment (T0), and at the end of the psychoeducation program (T1) was conducted through a series of χ^2^ tests, with a Yates correction when needed, and the binomial test, both for the experimental and control groups.

The correlations between QoL and the dysregulation of biorhythms in the experimental group were tested at T0 and T1 by Pearson’s R test.

## 3. Results

**Figure 1:**
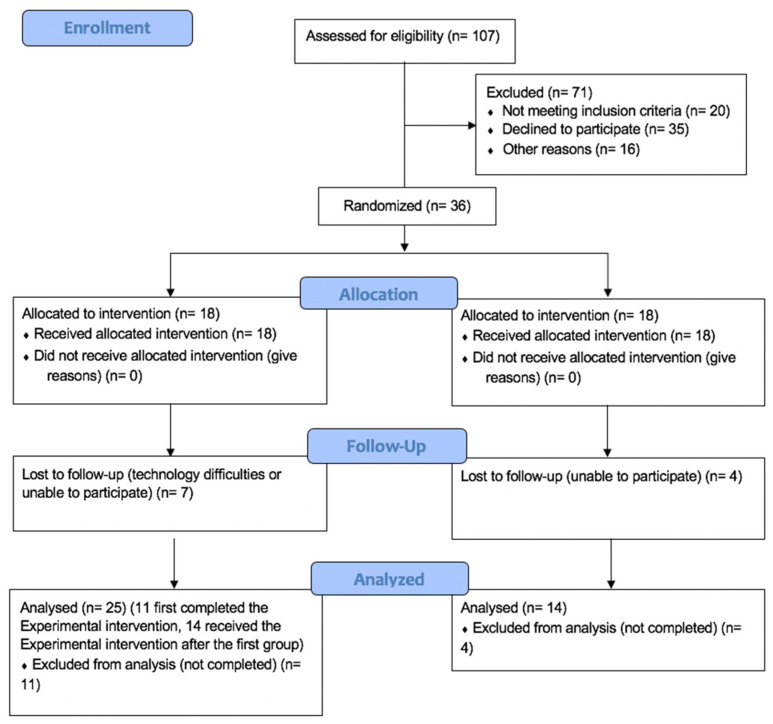
CONSORT flowchart extension for randomized trials.

As shown in the flowchart (Figure 1), and consistent with the inclusion and exclusion criteria, 107 subjects were initially contacted. Among them, 71 were excluded (20 did not meet the inclusion criteria, 35 declined to participate, and 16 did not respond) and 36 subjects were enrolled and randomized into two groups. Specifically, 18 were assigned to the experimental group, which received the e-health intervention, and 18 to the control group, and were initially placed on a waiting list and later crossed over to receive the experimental intervention. Before the crossover phase, seven subjects dropped out of the experimental group due to technology difficulties or inability to participate, and four from the control group were unable to participate.

Due to the crossover method, the experimental group finally included 25 subjects, while the control group included 14 subjects. 

As shown in Table 1, the experimental and control groups were homogeneous regarding the distributions of the variables “sex” and “age” (*p* = 0.986; *p* = 0.779). In both groups, 64% were females and 36% were males. The mean ± SD age was 48 ± 13.5 and 46.7 ± 13.6, in the experimental and control groups, respectively. 

As shown in Table 2, we found a statistically significant improvement in the QoL of the experimental group after treatment (χ^2^ = 6.480; *p* = 0.011). Even if it is without statistical significance, all the outcomes for the experimental group improved: the number of participants positive on the screening tests after treatment decreased (GAD7: χ^2^ = 0.802, *p* = 0.360; HAM-D: χ^2^ = 0.439, *p* = 0.508; BRIAN: χ^2^ = 0.439, *p* = 0.509). Furthermore, these improvements were more consistent in the experimental group (GAD7, −23%; HAM-D, −28.6%; SF12, −52.9%; BRIAN, −11.1%) than in the control group (GAD7, −18%; HAM-D, +33%; SF12, 125%; BRIAN, −11.1%). When a binomial test was performed, the probability of obtaining results for all five outcomes indicating a trend of better improvement in the experimental group was *p* < 0.0001.

Finally, as shown in Table 3, the correlations between QoL and the dysregulation of biorhythms in the experimental group were significant at T0 (R = 0.545; *p* = 0.0048) and T1 (R = 0.601; *p* = 0.0014).

## 4. Discussion

This study suggests that, during the extreme distress induced by the COVID-19 pandemic, an e-health, group psychoeducation intervention for people with BD improved the perception of QoL and, consequently, increased levels of optimism and coping skills. Although the intervention did not significantly improve any of the other clinical outcomes considered (anxiety and depressive symptoms, as well as rhythm dysregulation), these parameters, in line with the enhancement of QoL, suggest a trend toward greater improvement, or, in the case of depressive symptoms, less worsening of the experimental group compared to the control group. Given the presence of different clinical outcomes, the observed trend was statistically significant, suggesting that the failure to achieve a statistically significant difference in the comparisons may be attributed to a lack of study power, due to the small sample size. Supporting this thesis, the perception of QoL in the experimental group can be closely linked to the regulation of biological rhythms, both before and after the intervention, resulting in an improvement in QoL. This bidirectional relationship between these two parameters has been previously demonstrated [61], suggesting that an improvement in QoL can lead to better regulation of social rhythms and, consequently, the enhancement of clinical parameters [62,63]. This is unlike typical conditions, where symptoms and social functioning improve before the perception of QoL does over an extended period [52]. This aspect needs to be confirmed; however, it could be inferred in light of the unusual circumstances of the pandemic. This was particularly relevant during the COVID-19 pandemic, when individuals with significant vulnerability might have felt threatened and unable to manage the danger, which was exacerbated by a sense of loneliness. From this point of view, this study suggests that an e-health psychoeducational intervention could be effective for different reasons: (1) it reduced loneliness during the COVID-19 pandemic and lockdown; (2) it offered tools for the better management of BD, and was aligned with the mental health perspective that aims to reduce stigma (by placing the individual at the center of the care process and in the management of the disorder) and the evolutionary perspective that emphasizes social determinants in psychopathological processes [29,64,65,66]; (3) it offered information to participants on how to protect themselves from the pandemic and lower their risk. Consequently, this kind of intervention could make participants less passive; instead of waiting for the “inevitable catastrophe”, they become more responsible and proactive regarding risk management, and more optimistic. From this perspective, it is understandable why QoL is the parameter that improves the first. This study, consistent with other observations, shows that this approach to the crisis was not without positive aspects [66]. For example, the good accessibility of the e-health psychoeducation intervention could make it an easy tool to incorporate into daily practice that deserves to be strengthened and refined. 

The limitations of this study are associated with its inherently empirical and “in-the-field intervention” approach. These characteristics influenced this study’s design (necessarily crossover), the use of an open-label method, the sample size, and the adoption of straightforward and online-accessible assessment tools. Nevertheless, these constraints transformed this intervention into an exemplification of the “do-it-yourself” telemedicine approach that became firmly integrated into Italian healthcare practices in response to the COVID-19 pandemic. 

## 5. Conclusions

This study shows that, during periods of extreme distress, an e-health group psychoeducation intervention for individuals with BD could significantly improve their perception of their quality of life (QoL). The are several clinical implications, notably the increasing importance of developing personalized rehabilitative interventions capable of adapting to societal challenges during emergencies. Such accessible and adaptable interventions are crucial for ensuring continued access to effective care and support among vulnerable populations. However, in terms of research implications, these results should be validated through rigorous studies. Future research should prioritize robust methodologies, including double-blind randomized controlled trials with large sample sizes. These aspects could provide evidence that the intervention improves QoL among individuals with BD, and could enhance the reliability and generalizability of the findings.

## Figures and Tables

**Table 1:** Demographic characteristics (T0).

		EG	CG	*p*
SEX	F N (%)	16 (64%)	9 (64%)	0.986
	M N (%)	9 (36%)	5 (36%)	
AGE	N	25	14	0.779
	Mean ± SD	48 ± 13.5	46.7 ± 13.6	

F: female; M: male; EG: experimental group; CG: control group.

**Table 2:** Changes from T0 to T1 in the experimental and control groups about anxiety symptoms, depressive symptoms, QoL, and dysregulation of biorhythms.

	Anxiety Symptoms GAD 7 >7	χ^2^ *; *p*	Depressive Episode HAM-D >20	χ^2^ *; *p*	QoL SF-12 < 25	χ^2^ *; *p*	Rhythms Dysregulation BRIAN >33	χ^2^ *; *p*
EG T0	18/25	0.802	7/25	0.439	17/25	6.480	20/25	0.439
T1	15/25	*p* = 0.360	5/25	*p* = 0.508	8/25	*p* = 0.011	18/25	*p* = 0.509
% Differences	−23%		−28.6%		−52.9%		−11.1%	
CG T0	9/14	0.117	3/14	0.190	4/14	2.895	9/14	0.164
T1	11/14	*p* = 0.737	4/14	*p* = 0.663	9/14	*p* = 0.089	10/14	*p* = 0.686
% Differences	−18%		+33%		+125%		+11.1%	

*: with Yates correction when needed; EG: experimental group; CG: control group; T0: pretreatment; T1: after the end of psychoeducation program.

**Table 3:** Correlations between QoL and dysregulation of biorhythms in the experimental group.

	SF-12	BRIAN	R Pearson	*p*
T0 (Mean ± SD)	24.5 ± 7.64	44.2 ± 10.5	0.545	0.0048
T1 (Mean ± SD)	27.2 ± 8.25	43.4 ± 12.3	0.601	0.0014

T0: pretreatment; T1: after the end of psychoeducation program.

## Data Availability

To ensure confidentiality in compliance with European data protection laws, this study’s data were entered into a secure database and fully anonymized.

## References

[1] Carta M.G., Orrù G., Littera R., Firinu D., Chessa L., Cossu G., Primavera D., Del Giacco S., Tramontano E., Manocchio N. (2023). Comparing the Responses of Countries and National Health Systems to the COVID-19 Pandemic: A Critical Analysis with a Case-Report Series. Eur. Rev. Med. Pharmacol. Sci..

[2] Alhusban A., Alzoubi K.H., Al-Azzam S., Nuseir K.Q. (2022). Evaluation of Vulnerability Factors for Developing Stress and Depression Due to COVID-19 Spread and Its Associated Lockdown. Clin. Pract. Epidemiol. Ment. Health.

[3] Bellan M., Soddu D., Balbo P.E., Baricich A., Zeppegno P., Avanzi G.C., Baldon G., Bartolomei G., Battaglia M., Battistini S. (2021). Respiratory and Psychophysical Sequelae among Patients with COVID-19 Four Months after Hospital Discharge. JAMA Netw. Open.

[4] Di Fazio N., Morena D., Delogu G., Volonnino G., Manetti F., Padovano M., Scopetti M., Frati P., Fineschi V. (2022). Mental Health Consequences of COVID-19 Pandemic Period in the European Population: An Institutional Challenge. Int. J. Environ. Res. Public Health.

[5] Brooks S.K., Webster R.K., Smith L.E., Woodland L., Wessely S., Greenberg N., Rubin G.J. (2020). The Psychological Impact of Quarantine and How to Reduce It: Rapid Review of the Evidence. Lancet.

[6] Sher L. (2020). The Impact of the COVID-19 Pandemic on Suicide Rates. QJM.

[7] Alomari M.A., Khabour O.F., Alzoubi K.H., Aburub A. (2023). The Impact of COVID-19 Confinement on Reading Behavior. Clin. Pract. Epidemiol. Ment. Health.

[8] Vanhaecht K., Seys D., Bruyneel L., Cox B., Kaesemans G., Cloet M., Van Den Broeck K., Cools O., De Witte A., Lowet K. (2021). COVID-19 Is Having a Destructive Impact on Health-Care Workers’ Mental Well-Being. Int. J. Qual. Health Care.

[9] Moro M.F., Calamandrei G., Poli R., Di Mattei V., Perra A., Kurotschka P.K., Restrepo A., Romano F., La Torre G., Preti E. (2022). The Impact of the COVID-19 Pandemic on the Mental Health of Healthcare Workers in Italy: Analyzing the Role of Individual and Workplace-Level Factors in the Reopening Phase after Lockdown. Front. Psychiatry.

[10] Yassin A., Al-Mistarehi A.-H., Soudah O., Karasneh R., Al-Azzam S., Qarqash A.A., Khasawneh A.G., Alhayk K.A., Al Qawasmeh M., Kofahi R.M. (2022). Trends of Prevalence Estimates and Risk Factors of Depressive Symptoms among Healthcare Workers over One Year of the COVID-19 Pandemic. Clin. Pract. Epidemiol. Ment. Health.

[11] Wu Z., McGoogan J.M. (2020). Characteristics of and Important Lessons from the Coronavirus Disease 2019 (COVID-19) Outbreak in China: Summary of a Report of 72 314 Cases from the Chinese Center for Disease Control and Prevention. JAMA.

[12] Carta M.G., Bhugra D. (2022). From Stigma to Forgetfulness: The Rights of People with Psychosocial Disabilities in the New Middle Ages of the Covid Era. Int. J. Soc. Psychiatry.

[13] Novak P., Sanmartin M.X., Ali M.M., Chen J. (2021). Health Conditions Associated with Severe Illness from COVID-19 among Individuals with Serious Mental Illness. Psychiatr. Serv..

[14] Das-Munshi J., Bakolis I., Bécares L., Dyer J., Hotopf M., Ocloo J., Stewart R., Stuart R., Dregan A. (2023). Severe Mental Illness, Race/Ethnicity, Multimorbidity and Mortality Following COVID-19 Infection: Nationally Representative Cohort Study. Br. J. Psychiatry.

[15] Carta M.G., Ouali U., Perra A., Ben Cheikh Ahmed A., Boe L., Aissa A., Lorrai S., Cossu G., Aresti A., Preti A. (2021). Living with Bipolar Disorder in the Time of Covid-19: Biorhythms during the Severe Lockdown in Cagliari, Italy, and the Moderate Lockdown in Tunis, Tunisia. Front. Psychiatry.

[16] Felician J., Galvao F., Lefebvre M., Nourredine M., Peter-Derex L. (2022). Association of Delayed Sleep/Wake Rhythm with Depression during the First COVID-19 Lockdown in France. Nat. Sci. Sleep.

[17] Carvalho A.F., Firth J., Vieta E. (2020). Bipolar Disorder. N. Engl. J. Med..

[18] Sanchez-Moreno J., Martinez-Aran A., Vieta E. (2017). Treatment of Functional Impairment in Patients with Bipolar Disorder. Curr. Psychiatry Rep..

[19] Alonso J., Petukhova M., Vilagut G., Chatterji S., Heeringa S., Üstün T.B., Alhamzawi A.O., Viana M.C., Angermeyer M., Bromet E. (2011). Days out of Role Due to Common Physical and Mental Conditions: Results from the WHO World Mental Health Surveys. Mol. Psychiatry.

[20] Alloy L.B., Walsh R.F.L., Smith L.T., Maddox M.A., Olino T.M., Zee P.C., Nusslock R. (2023). Circadian, Reward, and Emotion Systems in Teens Prospective Longitudinal Study: Protocol Overview of an Integrative Reward-Circadian Rhythm Model of First Onset of Bipolar Spectrum Disorder in Adolescence. BMC Psychiatry.

[21] Carta M.G., Fornaro M., Minerba L., Pau M., Velluzzi F., Atzori L., Aviles Gonzalez C.I., Romano F., Littera R., Chessa L. (2022). Previous Functional Social and Behavioral Rhythms Affect Resilience to Covid-19-Related Stress among Old Adults. J. Public Health Res..

[22] Cossu G., Aviles Gonzalez C.I., Minerba L., Demontis R., Pau M., Velluzzi F., Ferreli C., Atzori L., Machado S., Fortin D. (2022). Exercise Improves Long-Term Social and Behavioral Rhythms in Older Adults: Did It Play a Role during the COVID-19 Lockdown?. J. Public Health Res..

[23] Giovanni Carta M., Kalcev G., Scano A., Aviles Gonzalez C.I., Ouali U., Pinna S., Carrà G., Romano F., Preti A., Orrù G. (2023). The Impact of MDQ Positivity on Quality of Life Impairment: Does It Support the Hypothesis of “Dysregulation of Mood, Energy, and Social Rhythms Syndrome” (DYMERS)?. J. Public Health Res..

[24] Carta M.G., Kalcev G., Scano A., Primavera D., Orrù G., Gureye O., Cossu G., Nardi A.E. (2022). Is Bipolar Disorder the Consequence of a Genetic Weakness or Not Having Correctly Used a Potential Adaptive Condition?. Brain Sci..

[25] Carta M.G., Kalcev G., Fornaro M., Pinna S., Gonzalez C.I.A., Nardi A.E., Primavera D. (2023). Does Screening for Bipolar Disorders Identify a “Dysregulation of Mood, Energy, and Social Rhythms Syndrome” (DYMERS)? A Heuristic Working Hypothesis. J. Clin. Med..

[26] Carta M.G., Kalcev G., Scano A., Pinna S., Gonzalez C.I.A., Nardi A.E., Orrù G., Primavera D. (2023). Screening, Genetic Variants, and Bipolar Disorders: Can Useful Hypotheses Arise from the Sum of Partial Failures?. Clin. Pract..

[27] Bianchi D., Lonigro A., Norcia A.D., Tata D.D., Pompili S., Zammuto M., Cannoni E., Longobardi E., Laghi F. (2022). A Model to Understand COVID-19 Preventive Behaviors in Young Adults: Health Locus of Control and Pandemic-Related Fear. J. Health Psychol..

[28] Aksenova E.I., Kamynina N.N., Metelskaya A.V., Shkrumyak A.R. (2021). On the Need for Psychological Support for Citizens in the Context of the COVID-19 Pandemic. Probl. Sotsialnoi. Gig. Zdravookhranenniiai. Istor. Med..

[29] de Siqueira Rotenberg L., Nascimento C., Cohab Khafif T., Silva Dias R., Lafer B. (2020). Psychological Therapies and Psychoeducational Recommendations for Bipolar Disorder Treatment during COVID-19 Pandemic. Bipolar Disord..

[30] Colom F., Vieta E., Scott J. (2006). Psychoeducation Manual for Bipolar Disorder.

[31] Chatterton M.L., Stockings E., Berk M., Barendregt J.J., Carter R., Mihalopoulos C. (2017). Psychosocial Therapies for the Adjunctive Treatment of Bipolar Disorder in Adults: Network Meta-Analysis. Br. J. Psychiatry.

[32] Zhao S., Sampson S., Xia J., Jayaram M.B. (2015). Psychoeducation (Brief) for People with Serious Mental Illness. Cochrane Libr..

[33] McGill C.W., Falloon I.R.H., Boyd J.L., Wood-Siverio C. (1983). Family Educational Intervention in the Treatment of Schizophrenia. Psychiatr. Serv..

[34] Bhugra D., Ventriglio A. (2023). Social Psychiatry and the NHS. Int. J. Soc. Psychiatry.

[35] Giusti L., Ussorio D., Salza A., Casacchia M., Roncone R. (2022). Easier Said than Done: The Challenge to Teach “Personal Recovery” to Mental Health Professionals through a Short, Targeted and Structured Training Programme. Community Ment. Health J..

[36] Rajkumar R.P. (2020). COVID-19 and Mental Health: A Review of the Existing Literature. Asian J. Psychiatr..

[37] Torous J., Keshavan M. (2020). COVID-19, Mobile Health and Serious Mental Illness. Schizophr. Res..

[38] Chinvararak C., Kirdchok P., Wonglertwisawakorn C., Pumjun P., Kerdcharoen N. (2024). Efficacy of Online Psychoeducation and Relaxation Training Program (OnPR) on Mental Health Problems in COVID-19 Patients: A Randomized Controlled Trial. Internet Interv..

[39] Carta M.G., Nardi A.E., Pinna S., Cossu G., Gureje O. (2023). Multidisciplinary Contributions towards an Evolutive Interpretation of Bipolar Disorders: Could It Be the Pathological Drift of a Potentially Adaptive Condition?. Rev. Bras. Psiquiatr..

[40] Rabelo J.L., Cruz B.F., Ferreira J.D.R., de Mattos Viana B., Barbosa I.G. (2021). Psychoeducation in Bipolar Disorder: A Systematic Review. World J. Psychiatry.

[41] Carta M.G., Nardi A.E., Bhugra D. (2021). New Technologies for Social Inclusion of People with Psychosocial Disabilities in the Era of COVID-19 and Beyond. Rev. Bras. Psiquiatr..

[42] Zhou X., Snoswell C.L., Harding L.E., Bambling M., Edirippulige S., Bai X., Smith A.C. (2020). The Role of Telehealth in Reducing the Mental Health Burden from COVID-19. Telemed. J. E. Health..

[43] Smith A.C., Thomas E., Snoswell C.L., Haydon H., Mehrotra A., Clemensen J., Caffery L.J. (2020). Telehealth for Global Emergencies: Implications for Coronavirus Disease 2019 (COVID-19). J. Telemed. Telecare.

[44] Schulz K.F., Altman D.G., Moher D., for the CONSORT Group (2010). CONSORT 2010 Statement: Updated Guidelines for Reporting Parallel Group Randomised Trials. BMJ.

[45] American Psychiatric Association (2013). Diagnostic and Statistical Manual of Mental Disorders: DSM-5.

[46] Spitzer R.L., Kroenke K., Williams J.B.W., Löwe B. (2006). A Brief Measure for Assessing Generalized Anxiety Disorder: The GAD-7. Arch. Intern. Med..

[47] Johnson S.U., Ulvenes P.G., Øktedalen T., Hoffart A. (2019). Psychometric Properties of the General Anxiety Disorder 7-Item (GAD-7) Scale in a Heterogeneous Psychiatric Sample. Front. Psychol..

[48] Cicchetti D.V. (1983). Reliability of Depression and Associated Clinical Symptoms. Arch. Gen. Psychiatry.

[49] Bech P., Allerup P., Larsen E.R., Csillag C., Licht R.W. (2014). The Hamilton Depression Scale (HAM-D) and the Montgomery–Åsberg Depression Scale (MADRS). A Psychometric Re-Analysis of the European Genome-Based Therapeutic Drugs for Depression Study Using Rasch Analysis. Psychiatry Res..

[50] Raimo S., Trojano L., Spitaleri D., Petretta V., Grossi D., Santangelo G. (2015). Psychometric Properties of the Hamilton Depression Rating Scale in Multiple Sclerosis. Qual. Life Res..

[51] Gandek B., Ware J.E., Aaronson N.K., Apolone G., Bjorner J.B., Brazier J.E., Bullinger M., Kaasa S., Leplege A., Prieto L. (1998). Cross-Validation of Item Selection and Scoring for the SF-12 Health Survey in Nine Countries. J. Clin. Epidemiol..

[52] Carta M.G., Aguglia E., Caraci F., Dell’Osso L., Di Sciascio G., Drago F., Del Giudice E., Faravelli C., Hardoy M.C., Lecca M.E. (2012). Quality of life and urban/rural living: Preliminary results of a community survey in Italy. Clin. Pract..

[53] Ruotolo I., Berardi A., Sellitto G., Panuccio F., Polimeni A., Valente D., Galeoto G. (2021). Criterion Validity and Reliability of SF-12 Health Survey Version 2 (SF-12v2) in a Student Population during COVID-19 Pandemic: A Cross-Sectional Study. Depress. Res. Treat..

[54] Moro M.F., Carta M.G., Pintus M., Pintus E., Melis R., Kapczinski F., Vieta E., Colom F. (2014). Validation of the Italian Version of the Biological Rhythms Interview of Assessment in Neuropsychiatry (BRIAN): Some Considerations on Its Screening Usefulness. Clin. Pract. Epidemiol. Ment. Health.

[55] Amendola S., Spensieri V., Hengartner M.P., Cerutti R. (2021). Mental Health of Italian Adults during COVID-19 Pandemic. Br. J. Health Psychol..

[56] Veltro F., Latte G., Pontarelli I., Pontarelli C., Nicchiniello I., Zappone L. (2022). Positive Impact of InteGRO, a New Salutogenic Psychoeducational Intervention, in Managing Covid-19 Pandemic and Lockdown Aftermath. Riv. Psichiatr..

[57] Perra A., Riccardo C.L., De Lorenzo V., De Marco E., Di Natale L., Kurotschka P.K., Preti A., Carta M.G. (2023). Fully Immersive Virtual Reality-Based Cognitive Remediation for Adults with Psychosocial Disabilities: A Systematic Scoping Review of Methods Intervention Gaps and Meta-Analysis of Published Effectiveness Studies. Int. J. Environ. Res. Public Health.

[58] Colom F., Lam D. (2005). Psychoeducation: Improving Outcomes in Bipolar Disorder. Eur. Psychiatry.

[59] Perra A., Galetti A., Zaccheddu R., Locci A., Piludu F., Preti A., Primavera D., Di Natale L., Nardi A.E., Kurotshka P.K. (2023). A Recovery-Oriented Program for People with Bipolar Disorder through Virtual Reality-Based Cognitive Remediation: Results of a Feasibility Randomized Clinical Trial. J. Clin. Med..

[60] Perra A., De Lorenzo V., Zaccheddu R., Locci A., Piludu F., Preti A., Di Natale L., Galetti A., Nardi A.E., Cossu G. (2022). Cognitive Remediation Virtual Reality Tool a Recovery-Oriented Project for People with Bipolar Disorder: Protocol of a Feasibility Randomized Clinical Trial. Clin. Pract. Epidemiol. Ment. Health.

[61] Carta M.G., Pintus E., Zaccheddu R., Callia O., Conti G., Aviles Gonzalez C.I., Minerba L., Demontis R., Pau M., Cocco E. (2022). Social and Behavioral Rhythms is Related to the Perception of Quality of Life in Old Adults. Open Psychol. J..

[62] Shahar S., Lynch S., Dornbush R., Klepacz L., Smiley A., Ferrando S.J. (2023). Frequency and Characteristics of Depression and Its Association with Diminished Quality of Life in a Cohort of Individuals with Post-Acute Sequelae of COVID-19. Neuropsychiatr. Dis. Treat..

[63] Primavera D., Aviles Gonzalez C.I., Romano F., Kalcev G., Pinna S., Minerba L., Scano A., Orrù G., Cossu G. (2023). Does the Response to a Stressful Condition in Older Adults with Life Rhythm Dysregulations Provide Evidence of the Existence of the “Dysregulation of Mood, Energy, and Social Rhythms Syndrome”?. Healthcare.

[64] Thomas E.C., Despeaux K.E., Drapalski A.L., Bennett M. (2018). Person-Oriented Recovery of Individuals with Serious Mental Illnesses: A Review and Meta-Analysis of Longitudinal Findings. Psychiatr. Serv..

[65] Kirkbride J.B., Anglin D.M., Colman I., Dykxhoorn J., Jones P.B., Patalay P., Pitman A., Soneson E., Steare T., Wright T. (2024). The Social Determinants of Mental Health and Disorder: Evidence, Prevention and Recommendations. World Psychiatry.

[66] Carta M., Sancassiani F., Ganassi R., Melis P., D’Oca S., Atzeni M., Velluzzi F., Ferreli C., Atzori L., Aviles-Gonzales C. (2022). Why Was the Perception of Human Rights Respect and Care Satisfaction so High in Users of Italian Mental Health Services during the COVID-19 Pandemic?. J. Clin. Med..

[67] (2013). World Medical Association Declaration of Helsinki: Ethical Principles for Medical Research Involving Human Subjects. JAMA.

